# Screening of a ScFv Antibody With High Affinity for Application in Human IFN-γ Immunoassay

**DOI:** 10.3389/fmicb.2018.00261

**Published:** 2018-03-07

**Authors:** Hang Yang, Yanfang Zhong, Juncheng Wang, Qinghong Zhang, Xiulan Li, Sumei Ling, Shihua Wang, Rongzhi Wang

**Affiliations:** Key Laboratory of Pathogenic Fungi and Mycotoxins of Fujian Province, Key Laboratory of Biopesticide and Chemical Biology of Education Ministry, School of Life Sciences, Fujian Agriculture and Forestry University, Fuzhou, China

**Keywords:** interferon gamma (IFN-γ), scFv, phage display, affinity, ic-ELISA, detection

## Abstract

Interferon gamma (IFN-γ), a signal proinflammatory cytokine secreted by immune cell, and plays a critical role in the pathogenesis and progression of many diseases. It has been regarded as an important marker for determination of disease-specific immune responses. Therefore, it is urgent to develop a feasible and accurate method to detect IFN-γ in clinic real blood samples. Until now, the immunoassay based on singe chain variable fragment (scFv) antibody for human IFN-γ is still not reported. In the present study, an scFv antibody named scFv-A8 with high specificity was obtained by phage display and biopanning, with the affinity 2.6 × 10^9^ L/mol. Maltose binding protein (MBP) was used to improve the solubility of scFv by inserting an linker DNA between scFv and MBP tag, and the resulted fusion protein (MBP-LK-scFv) has high solubility and antigen biding activity. The expressed and purified MBP-LK-scFv antibody was used to develop the indirect competitive enzyme-linked immunosorbent assay (ELISA) (ic-ELISA) for detection of human IFN-γ, and the result indicated that the linear range to detect IFN-γ was 6–60 pg/mL with IC_50_ of 25 pg/mL. The limit of detection was 2 pg/mL (1.3 fm), and the average recovery was 85.05%, further demonstrating that the detection method based on scFv has higher recovery and accuracy. Hence, the developed ic-ELISA can be used to detect IFN-γ in real samples, and it may be further provided a scientific basis for disease diagnosis.

## Introduction

To effective regulate immune response, would healing, and tissue regeneration, a large of different cytokines were released by immune cells, including T cells, phagocyte and NK cells (Liu et al., 2012). Interferon gamma (IFN-γ) is an important cytokine secreted mainly by immune cells under antigens stimulation, playing a key role in antiviral, antiproliferative, differentiation inducing, and immunoregulatory properties (Liu et al., 2010; Shi et al., 2017). IFN-γ is a small size of cytokine containing 146 amino acids in the maturated peptide, and it is also the sole member of the type 2 interferon family (Wu et al., 2017). Recently, IFN-γ detection was regarded as an important biomarker and employed to diagnose latent tuberculosis in clinic (Liu et al., 2012; Chegou et al., 2013; Olivieri et al., 2016). Some published papers have demonstrated that IFN-γ is required to prime macrophages in order to become fully activated and induce an efficient type I response in African trypanosome infections (Magez et al., 2002; Mansfield and Paulnock, 2005; Cnops et al., 2015). In addition, dysregulation of IFN-γ secretion is associated with various diseases, such as inflammatory disease (Zoller et al., 2011), human virus infections (Delannoy et al., 1999; Cimini et al., 2017), Breast tumors (Yuliatun et al., 2017), Latent trypanosome brucei gambiense infection (Ilboudo et al., 2016) and Early Lyme disease (Callister et al., 2016). It was also reported that IFN-γ was used as a novel therapy agent to treat idiopathic pulmonary fibrosis (IPF) (Wang J. et al., 2017). In view of the vital biological function of IFN-γ and important clinic detection significance, it is necessary to develop an accurate and feasible method to detect IFN-γ for investigating the vigor of the immune response as well as for the diagnosis of potential diseases (Zamani et al., 2017).

To date, some traditional techniques have been used to detect IFN-γ in real samples, including cyclometalated iridium (III) complex conductor (Miao et al., 2017), micropatterned aptamer-modified electrodes (Liu et al., 2012), DNA aptamer-based electrochemical biosensor (Liu et al., 2010) and ELISpot (Sanchez et al., 2014). Although these methods are effective and accurate for detection of IFN-γ, but they are often inconvenient, time-consuming, and need expensive equipment (Wang et al., 2016). Besides, the complex samples treatment and the process of data analysis have severally restricted their applications in target antigen detection (Ling et al., 2015b). The above situation indicates that these methods are not appropriate for IFN-γ detection in special environments that different to the laboratory. Enzyme-linked immunosorbent assay (ELISA) was becoming increasingly popular methods to detect specific antigen in real samples owing to its high sensitivity and accuracy, inspected visually result, and low cost (Wang et al., 2013, 2014a), and ELISA assay is more effective to detect larger numbers of samples one time that can be accomplished with conventional analyses (Wang et al., 2013).

Enzyme-linked immunosorbent assay detection based on monoclonal antibody has high affinity and accuracy, but tedious animal immunity and ethical concerns were insufficient for fast screening of satisfactory antibody (Ling et al., 2015a; He et al., 2016; Höglind et al., 2017). Compared to polyclonal antibody or monoclonal antibody, singe chain variable fragment (scFv) antibody can be easily manipulated using genetic engineering to modify their binding behavior, and to improve the affinity and specificity (Wang et al., 2013), which has widely attracted the research interests (Wang et al., 2015). In the present study, recombinant human IFN-γ protein was expressed and purified successfully in *Escherichia coli*, and the titer of antibody from the immunized mouse with the purified protein was monitored by indirect ELISA after four times injection. To screen a scFv antibody against IFN-γ, a recombinant library with large capacity was constructed. A high affinity scFv antibody was isolated and identified successfully after six rounds of biopanning, and the isolated scFv was specific to the IFN-γ antigen. At last, a competitive indirect ELISA was developed to detect IFN-γ based on this scFv antibody, providing a necessary basis for the effective immunoassay for IFN-γ.

## Materials and Methods

### Materials

All the strains used in this study were from Fujian Agriculture and Forestry University (Fujian, China). PCR ingredients and DNA restriction enzymes were purchased from Thermo Fisher Scientific (Waltham, MA, United States). Taq DNA polymerase and T4 DNA ligase were purchased from Takara biotechnology Co., Ltd. (Dalian, China). Anti maltose binding protein (MBP) tag monoclonal antibody was purchased from Abgent (San Diego, CA, United States), and HRP-labeled goat anti-mouse IgG was from Boster Biological Technology Co. (Wuhan, China). All other reagents used were of analytical reagent grade.

### Expression and Purification of IFN-γ

To obtain the recombinant IFN-γ antigen, the gene that encoding the IFN-γ was synthesized by Biosun Biotechnology company (Fuzhou, China) according to the DNA sequence. The amplified *IFN-*γ gene with *EcoR* I and *Hind* III restriction enzymatic sites was inserted into pET28a expression vector. The constructed vector pET28a-*IFN-*γ was transformed into *E. coli* BL21 (DE3) by electroporation, and the target protein was expressed through IPTG inducing (1 mM) when the culture reached to an OD_600_ of 0.8. After sonication and centrifugation, the collected supernatant was loaded into the Ni^2+^-NTA column for protein purification by affinity chromatography. The resulted protein was analyzed by SDS-PAGE, and the protein concentration was determined by using a bicinchoninic acid protein assay kit.

### Animal Immunization

Animal immunization was performed by standard procedure with minor modification (Wang et al., 2014a). The purified IFN-γ protein was used as an immunogen to immunize two Female *Balb/c* mice for generating antiserum with higher affinity. The IFN-γ antigen (0.2 mL, 100 μg) emulsified in Freund’s complete adjuvant was used for the first injection at multiple sites subcutaneously. Subsequently, about 2 weeks intervals, the IFN-γ antigen (0.1 mL, 50 μg) was emulsified with an equal volume of Freund’s incomplete adjuvant, and the resulted mixture was used to inject female Balb/c mice for generating of antiserum. The titer of serum was tested by ELISA after three times immunization (Wang et al., 2016).

### Construction of Phage Library Against IFN-γ

Total RNA was extracted from the spleen cells of immunized mice, and used to synthesize the cDNA by RT-PCR for construction of scFv antibody library (Wang et al., 2014a, 2016). The variable regions of heavy chain (*V*_H_) and light chain (*V*_L_) were amplified with the first strand cDNA as template through the primary PCR amplification. A special linker DNA fragment encoding a short flexible peptide, (Gly4Ser)3 was used to assemble scFv gene fragments by overlap extension PCR (SOE-PCR) (molecular ratio of VH to VL to linker DNA is 3:3:1). The assembled scFv fragments were digested, and cloned into the phage plasmid pCANTAB-5E, and the ligated mixture was transformed into *E. coli* TG1 cells by electroporation. Then, the transformed cells were transferred into separate tubes containing 1 mL of LB-AG medium and incubated at 37°C for 45 min with shaking, and 10 μL of transformed cells were took out from the separate tubes and plated onto the SOB-AG plates with incubation at 37°C for overnight. The colony-forming unit was counted, and the capacity of constructed library was calculated according to the dilution ratio. The positive rate and diversity were determined by PCR and DNA sequence.

### Bio-Panning of ScFv Clones Against IFN-γ

To further screen scFv clones with high affinity against IFN-γ from the constructed library, bio-panning was performed as described (Rahbarnia et al., 2016). To enhance the efficiency of biopanning, the phage particles displaying scFv were precipitated with polyethylene glycol (PEG/NaCl) on ice for 1 h and collected by centrifugation at 10,000 *g* for 20 min at 4°C. A 96-wells micro titer plate was coated with IFN-γ antigen diluted to 2.5 μg/mL in PBS (100 μL/well), and incubated at 4°C for overnight. At the same time, a negative control was performed (uncoated with detection antigen). The plate was washing with PBS for three times and blocked with PBS containing 4% non-fat milk. The diluted recombinant phage particles were added into the plate (100 μL/well), and then the plate was incubated at 37°C for 2 h. The plate was washed 10 times with PBS and 10 times with PBS containing 0.05% Tween-20 to remove the unbound phages. Phage particles that specifically bind to IFN-γ were eluted with 10 mL of triethylamine for 10 min, and then 10 mL of Tris-HCl (pH 7.4) was added into the wells to neutralize the reaction. The log phase *E. coil* TG1 cells were infected with the eluted phages, and then plated onto SOB-AG plates for screening of individual colonies. The biopanning process was repeated for six rounds (Wang et al., 2014a).

### Screening of Clones With High Binding Activity From Enriched Clones

After four rounds of panning, 100 clones from different plates were picked randomly to culture individually with helper M_13_KO_7_ for phage ELISA analysis. To detect the binding activities of enriched clones, the phage ELISA was carried out. Briefly, the prepared phage was added into the pre-coated 96-wells ELISA plates for incubation at 37°C for 2 h. After washing 10 times with PBST and PBS directly, the binding phage was tested with an HRP-labeled anti-M13 antibody at a 1: 4000 dilution (Wang et al., 2014a).

### Construction of Expression Vectors for ScFv

The positive scFv clone with the highest binding activity was sequenced, and specific primers were used to amplify the target scFv gene. pET28a(+), pBD-*mbp*, and pBD-*mbp-linker* vectors were used for construction of expression vectors with the scFv proteins fused to His tag, MBP-His tags and MBP-Lk-His tags, respectively (Wang et al., 2016). The process of construction was same as the section “Expression and Purification of IFN-γ.”

### Expression and Soluble Analysis of ScFv Antibodies

For protein expression, the recombinant plasmids were transformed into *E. coli* BL21 by electroporation, and the target protein was expressed via IPTG inducing (1 mM) for overnight at 16°C when the culture reached to an OD_600_ of 0.8, and then the pellet was harvested by centrifugation. To further test the solubility of culture, three different derived cultures were adjusted to the same concentration for samples treatment, and the resulted proteins were analyzed by SDS-PAGE.

### Purification and Identification of Anti-IFN-γ Antibodies

The purification of the expressed anti-IFN-γ scFv was performed using Ni^2+^ affinity chromatography, and the detailed steps were same as the above protein purification. The activity of the purified scFv products was determined by ELISA. The purified IFN-γ antigen was used to coat the 96 wells plates for overnight at 4°C. After blocking and washing, the purified scFv products were added to the reaction wells and incubated for 2 h at 37°C. Then, the anti-MBP tag antibody was added to the reaction wells for reaction, and the binding activity of the purified scFv antibodies was detected by using a HRP-conjugated goat anti-mouse IgG antibody. The enzyme reaction was then performed using tetramethylbenzidine (TMB) as the substrate, and the color development was stopped by adding 2 M H_2_SO_4_. The absorbance at 450 nm was measured using a microplate reader (Wang et al., 2014b). To further confirm the binding activity of MBP-linker-scFv, western blotting was performed as described with minor modifications (Wang et al., 2013). Recombinant IFN-γ antigen was transformed onto a polyvinylidene difluoride (PVDF) membrane from SDS-PAGE gel, and the remaining steps were the same as the ELISA assay. Signals were visualized by enhanced chemiluminescence (ECL).

### Specificity and Affinity Determination

To determine the specificity of the expressed anti-IFN-γ scFv antibody (MBP-LK-scFv), ELISA was performed as described (Ling et al., 2014). Different protein antigens such as BSA, KLH, PDPN, OVA, HAS, and PBSM were diluted and coated in 96-well plates (1 μg/mL). The steps of ELISA were same as the above. To further determine the affinity of MBP-LK-scFv, the affinity of purified antibody (MBP-LK-scFv) was analyzed by ELISA with different antigen and antibody concentration. The affinity constant (*K*_aff_) of the antibody (MBP-LK-scFv) against IFN-γ was detected using the previous formula (Wang et al., 2012).

### Establishment of ic-ELISA for IFN-γ Assay

To develop a feasible method to detect IFN-γ, indirect competitive ELISA (ic-ELISA) was performed. The standard IFN-γ antigen was used as the competitive antigen and reacted with the purified MBP-linker-scFv (0.3 μg/mL) at 37°C for 2 h, and other ELISA steps corresponded to the steps described above. The data are presented as means and SDs for three separate experiments (Wang R. et al., 2017). To further detect the limit of detection (LOD), indirect competition ELISA was established according to previous researchers (Wang et al., 2016; Wang R. et al., 2017) with minor modification. Simply, 50 μL of standard IFN-γ antigen with different concentrations was mixed with equal volume of antibody. After incubation at 37°C for 30 min, the mixture was added into the reaction wells of IFN-γ antigen pre-coated plates, and incubated at 37°C for 1 h.

### Simulated and Real Samples Detection

The intra- and inter-assay were used to determine the repeatability, the recovery of sample, and accuracy of ic-ELISA. The average recovery of ic-ELISA was analyzed by addition of different concentrations of IFN-γ in samples that no IFN-γ antigen residual (Wang R. et al., 2017). In this study, the mean recovery and coefficient of variation (CV%) values of picked samples with different concentrations of IFN-γ (6.64, 12.8, 25.6, and 51.2 pg/mL) were detected at least three times. Meanwhile, the ic-ELISA was used to detect IFN-γ antigen in real samples after PBS treatment, and the concentration of IFN-γ was calculated according to the resulted standard curve. To ensure the accuracy of ic-ELISA, all the real samples were also tested by ELISA using a monoclonal antibody against IFN-γ as control.

## Results

### Expression and Purification of IFN-γ and Animal Immunization

**FIGURE 1:**
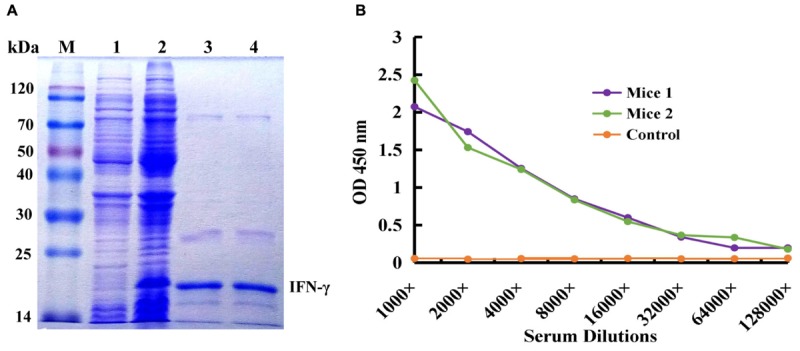
Expression and purification of human IFN-γ in *Escherichia coli* and animal immunization. **(A)** Expression and purification of human IFN-γ. Lane M: Protein Markers; lane 1: the expressed total protein of pET28a/BL21(DE3) as negative control; lane 2: the expressed total protein of pET28a-*ifn-*γ/BL21(DE3); lane 3, 4: the purified protein of IFN-γ-His6. **(B)** Titer determination of immunized mouse.

IFN-γ-His6 fusion protein (antigen) was expressed by IPTG inducing and purified by Ni^2+^ column chromatography successfully, and the result was showed in **Figure 1A**. The molecular weight of target protein was about 20 kDa, and it is correspond with the theoretical value. The purified recombinant IFN-γ protein was used as an immunogen to immune the mice for activation of immune response, and the titer of antiserum extracted from immunized mice was tested by ELISA. As shown in **Figure 1B**, compared to the control mice, mice 1 and mice 2 showed higher antiserum titer (reaching 1:8000), indicating that the immunized mice had high anti-IFN-γ antibody titer, and this could be used for further construction of immunized scFv library and bio-panning of specific scFv antibody against IFN-γ.

### Construction of ScFv Library and Bio-Panning of ScFv Clones Against IFN-γ

**FIGURE 2:**
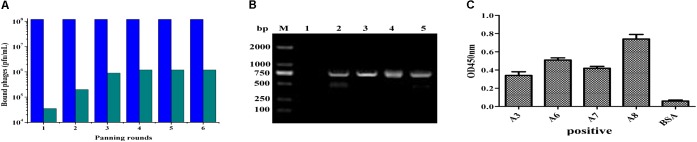
Construction and biopanning of scFv clones against IFN-γ. **(A)** The input and output of recombinant phage. The blue pillar indicated the input of recombinant phage, and the green pillar indicated the output of recombinant phage. **(B)** The amplified PCR results of selected scFv clones from biopanning result (M: DL-2000 DNA markers; lane 1: negative control; lane 2–5: the PCR products of scFv clones). **(C)** The ELISA result of the selected scFv clones. Here, the results showed the four scFv antibody clones with strong binding activity to IFN-γ antigen.

After amplification of *V*_H_ and *V*_L_ genes with cDNA as template by PCR, the scFv fragments were assembled and amplified by using SOE-PCR through a flexible linker DNA. The assembled scFv products were digested and inserted into the phage plasmid pCANTAB-5E, generating an immunized scFv gene library comprised of 2.9 × 10^8^ independent clones. The result from bio-panning demonstrated that the titer of the eluted phages after each round of panning was increased distinctly, and maintained a stable level (about 10^6^ pfu/mL) after three rounds of biopanning (**Figure 2A**), indicating that more specific antibodies bound to the IFN-γ antigen coated on the plates in the process of bio-panning. Then, several clones were selected randomly and identified by bacterial PCR, and all the clones were positive in PCR (**Figure 2B**). After six rounds of bio-panning, four clones that displaying strong binding activity to IFN-γ antigen were screened out by phage ELISA, and they were named scFv-A3, scFv-A6, scFv-A7, and scFv-A8, respectively (**Figure 2C**). On the basis of the above result, the scFv-A8 with the highest binding activity was selected for further research.

### Soluble Expression of ScFv

**FIGURE 3:**
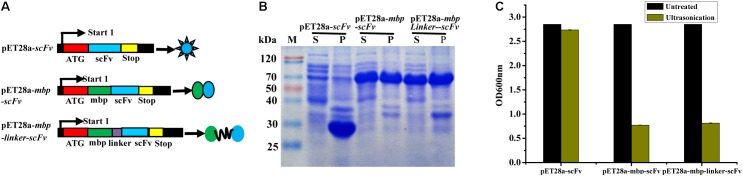
Construction and expression of different scFv fusion proteins against IFN-γ. **(A)** Constructs of scFv with different fusion formats used in this study. MBP, maltose binding protein; Sotp, stop codon; scFv, single chain fragment. **(B)** Soluble analysis of the expressed protein. S, the composition of soluble cell lysates; P, the composition of insoluble cell lysates. **(C)** Cell lysates analysis by OD_600_ nm test.

In this study, three different fusion expression vectors, pET28a-*scFv*, pET28a-*mbp-scFv*, and pET28a-*mbp-linker-scFv* were constructed successfully (**Figure 3A**), and transformed into *E. coli* BL21 (DE3) to investigate their effects on solubility of the expressed scFv antibody. As shown in **Figure 3B**, all the target scFv fusion proteins were expressed successfully. In comparison to the scFv-His6 fusion, MBP-scFv and MBP-linker-scFv fusion expression have similar protein solubility in supernatant, while a large portion of scFv proteins was expressed in a form of inclusion body in scFv-His6 fusion expression. This case was further confirmed by the measure of absorbance at OD_600__nm_ (**Figure 3C**). The above results indicated that MBP fusion expression was suitable for improving the solubility of scFv expression.

### Purification and Immunoassay of ScFv Fusion Proteins Against IFN-γ

**FIGURE 4:**
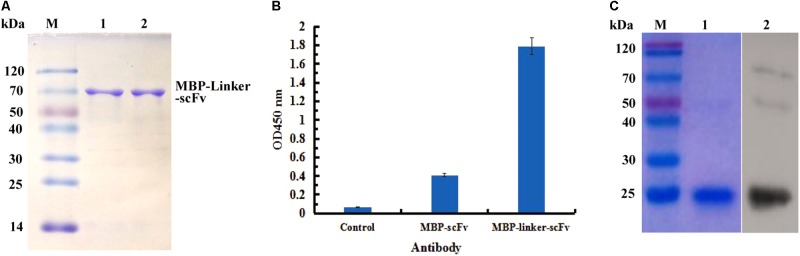
Purification and immunoassay of scFv fusion proteins against IFN-γ. **(A)** SDS-PAGE assay of the purified scFv proteins. Lane M: protien markers; lanes 1–3: the purified protein of scFv, MBP-scFv and MBP-Linker-scFv, respectively. **(B)** ELISA determination. IFN-γ were coated on 96-well plates in triplicate, and the purified scFv proteins were added to the reaction wells after blocking and washing, respectively. The binding activities of four purified proteins were determined using an anti-MBP tag antibody. **(C)** Western blot analysis of the binding activity of MBP-Linker-scFv to IFN-γ antigen. Left panel: SDS-PAGE result for IFN-γ; right panel: western blotting results; lane 2: IFN-γ band at 20 kDa bound by MBP-Linker-scFv.

In view of the solubility of protein, the expressed products derived from MBP fusion expression formats were purified successfully by affinity chromatography, and the purified scFv protein was analyzed by SDS-PAGE (**Figure 4A**). To determine the function of two different MBP fusion formats, the binding activities of the two purified proteins to IFN-γ antigen were assessed by ELISA. As seen in **Figure 4B**, the purified protein derived from the MBP-Lk-scFv fusion expression showed the highest binding activity to IFN-γ antigen, whereas the binding activity of MBP-scFv fusion protein was barely satisfactory. Those above results demonstrated that the MBP-scFv fusion expression increases the scFv solubility, and linker (Lk) between the scFv and MBP improves the binding activity of scFv. To further confirm the binding activity of MBP-Lk-scFv, western blotting was performed. A clear band at 20 kDa was revealed after imaging and photographic fixing (**Figure 4C**), indicating that the MBP-Lk-scFv effectively bound to the standard IFN-γ antigen immobilized on the PVDF membrane.

### Specificity Analysis and Affinity Determination of MBP-Lk-ScFv

**FIGURE 5:**
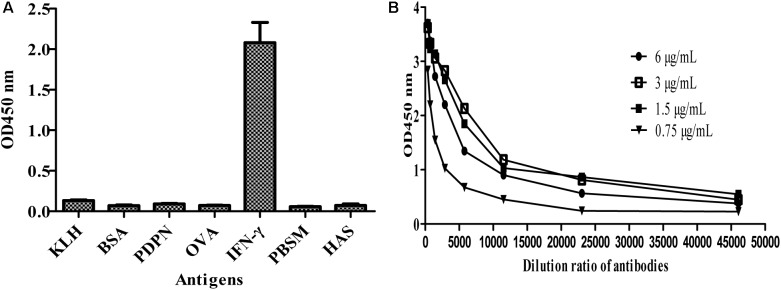
Specificity and affinity determination of scFv against IFN-γ. **(A)** Specificity analysis of MBP-Linker-scFv. Cross reactivity of MBP-Liner-scFv to other antigens was tested by iELISA. **(B)** Affinity determination of MBP-Linker-scFv. Four different concentrations of MBP-Linker-scFv were used to determine the affinity of MBP-Linker-scFv by ELISA, and the measured data were used for the quantitative determination of the affinity constant of MBP-Linker-scFv.

To further test the specificity of MBP-Lk-scFv to IFN-γ antigen, ELISA was carried out. As shown in **Figure 5A**, the purified MBP-LK-scFv was specific to IFN-γ antigen, and no cross-binding was observed to other antigens. Meanwhile, ELISA was used to determine the affinity of MBP-Lk-scFv, and the measured data were used for the quantitative determination of the affinity constant. The calculated affinity constant of MBP-Lk-scFv was 2.6 × 10^9^ L/mol (**Figure 5B**), belonging to a high affinity antibody. These results indicated that the expressed and purified MBP-Lk-scFv has high affinity and specific to IFN-γ, and could be used as an antibody reagent to detect IFN-γ.

### Sensitivity Determination and Standard Curve

**FIGURE 6:**
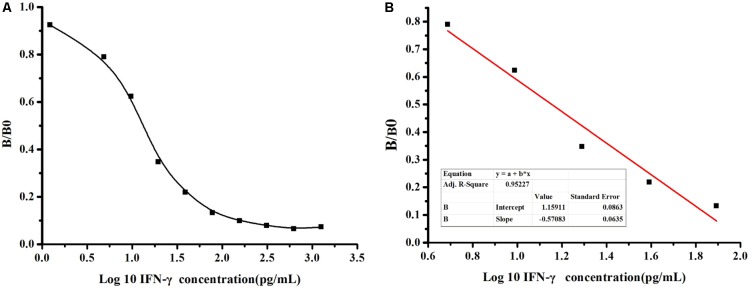
Sensitivity analysis of anti-IFN-γ-scFv. **(A)** The standard curve was carried out by ic-ELISA. The data obtained in the presence of various inhibitor and without inhibitor are referred to as B and B0, respectively. Standard curves were generated by plotting the inhibition percentage (B/B0) versus the log of three inhibitor concentrations. **(B)** The linear equation is *y* = –0.57x+1.159, with a correction coefficient (*R*^2^) of 0.9522.

To further determinate the sensitivity of detection based on MBP-Lk-scFv, ic-ELISA was performed to develop a standard curve for IFN-γ detection. The relationship between concentrations of IFN-γ and inhibition value was analyzed using Microcal Originpro 8.1. The half inhibitory concentration (IC_50_) of IFN-γ binding to scFv-5A10 was 25 pg/mL, where the linear range to detect IFN-γ was 6 ∼ 60 pg/mL, which defined as the concentration of CIT toward from 20 to 80% inhibition, and the LOD was 2 pg/mL (**Figure 6A**). The linear equation is *y* = -0.57x+1.159, with a correlation coefficient (*R*^2^) of 0.9522 (**Figure 6B**).

### Simulated and Real Samples Detection

In this study, the recovery of detection was analyzed based on the standard curve by ic-ELISA, and standard IFN-γ antigen was added to dilution buffer with different concentrations. The result showed that the recovery of detection ranged from 78.46 to 94.04%, with an average of 87.45%, and the variation coefficient was 1.18 ∼ 7.48% (average 3.76%) in the intra-assay. The recovery rate ranged from 77.71 to 90.89% with an average of 85.05%, and the variation coefficient was 1.27 ∼ 7.94% (average 4.075%) in the inter-assay (**Table 1**). The above result demonstrated that this assay had high repeatability and accuracy and it could be used for quantitative detection of IFN-γ. At last, two different real samples (PBS and ddH_2_O) that non-spiked IFN-γ were used for quantitative detection of IFN-γ by ic-ELISA. All the real samples were tested by ic-ELISA using a monoclonal antibody against IFN-γ as control for identification the existence of IFN-γ in real samples. As shown in **Table 2**, IFN-γ was not detected in these samples.

**Table 1:** The recovery and coefficient of variation (CV) detection in spiked samples.

	Intra-assay (*n* = 3)			Inter-assay (*n* = 3)		
Spiked level (pg/mL)	Measured (pg/mL)	Recovery (%)	CV (%)	Measured (pg/mL)	Recovery (%)	CV (%)
51.2	48.15 ± 0.57	94.04	1.18	46.54 ± 0.59	90.89	1.27
25.6	23.27 ± 0.52	90.89	2.23	22.39 ± 0.57	87.46	2.54
12.8	11.06 ± 0.46	86.40	4.16	10.77 ± 0.49	84.14	4.55
6.64	5.21 ± 0.39	78.46	7.48	5.16 ± 0.41	77.71	7.94
Average		87.45	3.76		85.05	4.075

**Table 2:** Enzyme-linked immunosorbent assay (ELISA) result for IFN-γ detection in real samples.

Sample	OD 450 nm value	Detection results
PBS	1.061	-a
ddH_2_O	1.058	-a

*-a means that no IFN-γ was detected out in real samples.*

## Discussion

Interferon gamma, an important regulator of immune responses that mainly produced by multiple types of immune cells, plays an essential role in immuno-modulation in infectious diseases (Schroder et al., 2004; Cnops et al., 2015; Wu et al., 2017). Hence, it is critical to develop an accurate method to detect IFN-γ secretion in real samples. At present, some different kinds of methods based on electrochemical detection (ECD) and ELISA are used to IFN-γ (Liu et al., 2010; Sanchez et al., 2014; Miao et al., 2017), but the above method based on ECD is improper for the actual sample detection because complex samples treatment and need expensive equipment/professional technicians. To date, immunoassays based on different kinds of antibodies, such as poly-clonal antibody, monoclonal antibody, and genetic engineering antibody, have also been used widely in immunological detection of target protein and pathogens in samples (Wang et al., 2012, 2014b, 2016; Saeed et al., 2017). scFv is an typical representative of the genetic engineering antibody. Compared to monoclonal antibody, the mainly advantages of scFv are low cost, genetically manipulation, consistent with genotype and phenotype and fast screening, and those prominent advantages greatly facilitated its application in biological target molecular (Wang et al., 2012; Ling et al., 2014; Saeed et al., 2017). Combined with the significance and clinical role of IFN-γ, it is a great choice for developing of immunoassay based on scFv to detect IFN-γ sensitively in samples. In this study, we developed a feasible method based on scFv antibody for the first time to detect IFN-γ in real samples. A 2.9 × 10^8^ capacity phage library was constructed by SOE-PCR to screen a high affinity scFv antibody against IFN-γ. After six rounds bio-panning, the isolated scFv-A8 has high binding activity to IFN-γ antigen, with an affinity of 2.6 × 10^9^ L/mol. The result indicated that the scFv-A8 obtained from phage library has high specificity and affinity that can be used for IFN-γ detection, and the developed method had high repeatability and accuracy.

Soluble expression of a functional scFv antibody is still a bottleneck problem faced in the present study. To find the optimal expression format for scFv, three different formats of fusion expression vectors were constructed. pET28a-*scFv* is the basic vector for scFv expression, while pET28a-*mbp-scFv* contains a mbp tag in N-terminals. pET28a-*mbp-linker-scFv* contains a flexible linker DNA between mbp tag and scFv gene. The irrefutable fact is that the absorbance of culture from MBP-scFv and MBP-linker-scFv fusion expression were clearly different to the scFv-His fusion after treatment by ultrasonication (data not shown), and this is consistent with our prediction before the test. The result from SDS-PAGE (**Figure 3B**) also demonstrated that the MBP-scFv and MBP-LK-scFv showed similar and higher solubility than the scFv derived from pET28a-*scFv*, further showing that MBP is an effective tag to enhance the solubility of target protein (Nallamsetty and Waugh, 2006; Raran-Kurussi and Waugh, 2017). In our previously study, different scFv fusion formats were used to improve the solubility and affinity of scFv, including TRx fusion and co-expression with molecular chaperon Skp. Compared to MBP fusion protein, the solubilities of scFv fusion proteins were enhanced after expression, however, the affinity from those scFv were unsatisfactory (Wang et al., 2013, 2016; Raran-Kurussi and Waugh, 2017). The result also indicated that MBP fusion can only increase the solubility of protein, but cannot improve the binding activity of scFv, and this fusion format is not suitable for expression of functional scFv. In contrast, MBP-LK-scFv had the highest binding activity to IFN-γ in ELISA, and this phenomenon stated clearly that the inserted linker DNA is critical for the folding of target protein. This is reasoned that the insertion of linker DNA may effectively reduce the interference of folding between MBP and scFv on the space (Wang et al., 2016).

Until now, immunoassay based on scFv antibody for IFN-γ detection in samples has not been reported. In this study, a positive scFv clone with the affinity of 2.6 × 10^9^ L/mol, named scFv-A8 was screened successfully by phage display and bio-panning, and the resulted scFv antibody was used for developing of immunoassay to detect IFN-γ in real samples. Compared to the ECD method, the developed method based on ic-ELISA has high specificity and accuracy, and the principal advantages of this method are simple, quick, efficient, and does not require sophisticated instruments and tedious sample pre-treatments. Ic-ELISA indicated that the linear range to detect IFN-γ was 6–60 pg/mL with IC_50_ of 25 pg/mL, and the LOD was 1.3 fm, which is lower than any other LOD reported previously (Liu et al., 2010; Miao et al., 2017).

## Conclusion

This screened anti-IFN-γ scFv with high specificity and affinity, and the developed ic-ELISA were feasible to determine and quantify the IFN-γ antigen in real samples. To our knowledge, this is the first time to develop an immunoassay based on scFv antibody for IFN-γ detection. Hence, the developed ic-ELISA method may be laid the foundation for the IFN-γ diagnosis and drug development, and this method will be provided a reference for infectious disease prediction and cancer research.

## Ethics Statement

This study was performed according to Principles of laboratory animal care were followed and all procedures were conducted according to the guidelines established by the National Institutes of Health, and every effort was made to minimize suffering. This study was approved by the Animal Experiment Committee of Fujian Agriculture and Forestry University.

## Author Contributions

SW and RW conceived the idea and designed the experiments. HY, YZ, JW, QZ, XL, and SL performed all the experiments. HY and RW analyzed all the data and wrote the manuscript. All authors reviewed the manuscript.

## Conflict of Interest Statement

The authors declare that the research was conducted in the absence of any commercial or financial relationships that could be construed as a potential conflict of interest.
